# Selection Signal Analysis Reveals Hainan Yellow Cattle Are Being Selectively Bred for Heat Tolerance

**DOI:** 10.3390/ani14050775

**Published:** 2024-02-29

**Authors:** Liuhao Wang, Xuehao Yan, Hongfen Wu, Feifan Wang, Ziqi Zhong, Gang Zheng, Qian Xiao, Kebang Wu, Wei Na

**Affiliations:** School of Tropical Agriculture and Forestry, Hainan University, Haikou 570228, China

**Keywords:** Hainan yellow cattle, selection signature, heat tolerance

## Abstract

**Simple Summary:**

Preserving genetic diversity is of significant importance for the sustainable development of livestock farming. Due to the unique environment in Hainan province, Hainan yellow cattle exhibit excellent heat tolerance and adaptability. Signal analysis of selection can provide valuable information for the conservation and utilization of this breed. Therefore, this study aims to compare the whole-genome sequencing data of Hainan yellow cattle with four other cattle breeds. Through the analysis of selection features, some candidate genes related to heat stress were identified.

**Abstract:**

Hainan yellow cattle are indigenous Zebu cattle from southern China known for their tolerance of heat and strong resistance to disease. Generations of adaptation to the tropical environment of southern China and decades of artificial breeding have left identifiable selection signals in their genomic makeup. However, information on the selection signatures of Hainan yellow cattle is scarce. Herein, we compared the genomes of Hainan yellow cattle with those of Zebu, Qinchuan, Nanyang, and Yanbian cattle breeds by the composite likelihood ratio method (CLR), Tajima’s D method, and identifying runs of homozygosity (ROHs), each of which may provide evidence of the genes responsible for heat tolerance in Hainan yellow cattle. The results showed that 5210, 1972, and 1290 single nucleotide polymorphisms (SNPs) were screened by the CLR method, Tajima’s D method, and ROH method, respectively. A total of 453, 450, and 325 genes, respectively, were identified near these SNPs. These genes were significantly enriched in 65 Gene Ontology (GO) functional terms and 11 Kyoto Encyclopedia of Genes and Genomes (KEGG) pathways (corrected *p* < 0.05). Five genes—Adenosylhomocysteinase-like 2, DnaJ heat shock protein family (Hsp40) member C3, heat shock protein family A (Hsp70) member 1A, CD53 molecule, and zinc finger and BTB domain containing 12—were recognized as candidate genes associated with heat tolerance. After further functional verification of these genes, the research results may benefit the understanding of the genetic mechanism of the heat tolerance in Hainan yellow cattle, which lay the foundation for subsequent studies on heat stress in this breed.

## 1. Introduction

The excessive emission of carbon dioxide leads to a global rise in temperature [1]. Global climate change has caused a series of problems, which have the biggest and most direct impact on agricultural production, especially crop production and food security as the main body of agriculture. The sustainability of livestock production systems is also significantly influenced by climate change. Cattle are one of the world’s important economically domesticated animals. In tropical and subtropical regions, they often experience stress due to high temperatures, impacting their growth rate, milk production, and reproductive performance [2,3,4,5,6]. Concerns over heat stress impacting cattle health will only rise as global temperatures increase. Obviously, minimizing the impact of heat stress on the production of cattle is an urgent issue in the cattle industry.

The characteristic cattle species in China are known as yellow cattle, and there are 55 native yellow cattle breeds in China. Native yellow cattle are often divided into three populations based on their relative distribution: northern yellow cattle, central yellow cattle, and southern yellow cattle. Hainan yellow cattle, a representative breed of southern yellow cattle, is praised for its associated heat resistance, all while producing high-quality meat [7]. The research on the high-temperature resistance of Hainan yellow cattle is basically blank. Selection signal analysis is a widely used method for identifying genetic variation underpinning breed-specific traits in livestock, including traits related to environmental adaptation [8]. The positively selected features in the genome serve as markers of adaptation, revealing ongoing responses to environmental changes throughout the population’s evolutionary process. Detecting these selected features can help conservation scientists and land managers understand how in gene regulation, the gene expression, protein synthesis, structure, and function might relate to cattle economic traits. For example, some studies have confirmed the presence of genetic variability for heat tolerance in cattle livestock [9,10,11]. Certain livestock breeds are adapted to hot and humid environments, and the genetic genes of these breeds can play a role in changing climates. The expression of some genes has been identified as useful biomarkers for heat stress. However, despite a general appreciation for the heat tolerance of Hainan yellow cattle, there are currently no reports on selection signals relating to years of past management. Herein, to explore the unique capacity of Hainan yellow cattle to cope with heat stress, we used composite likelihood ratio tests (the CLR method), Tajima’s D test, and ‘Runs of Homozygosity’ (ROHs—areas with contiguous homozygous regions in a genome associated with inbreeding) to analyze the selective signal of heat-resistant traits and screen candidate genes related to heat-resistant traits. The results can provide us with a better understanding of genetic variation related to heat-related traits, and further elucidate the genetic mechanism underpinning the heat tolerance associated with Hainan yellow cattle. At the same time, it provides a theoretical basis for the subsequent research on heat stress of other breeds of cattle.

## 2. Material and Methods

### 2.1. Animals

In this study, we collected data on 104 healthy Hainan yellow cattle with the age of 18–36 months and the weight of 200–300 kg. A total of 86 Hainan yellow cattle were gathered from Chengmai Cattle Farm of Hainan Huiniu Agricultural Technology Co., Ltd. (ChengMai, China) and 18 Hainan yellow cattle were collected from Yunlong Cattle Farm in Haikou. Data from 26 cattle of other breeds were obtained from NCBI for the comparative analysis of selection signals. The inclusion of data from these different breeds allows for a comparative analysis of selection signals, providing a broader perspective on genetic variations and adaptations among different cattle breeds. This diverse dataset enhances the robustness of this study’s findings and allows researchers to identify specific genetic traits unique to Hainan yellow cattle in comparison to other breeds. In our analysis, we included data from 9 Zebu cattle (ZB), 2 Qinchuan cattle (QC), 9 Yanbian cattle (YB), and 6 Nanyang cattle (NY) (Supplementary Table S1).

### 2.2. DNA Sample Preparation and Whole-Genome Sequencing

After collection of blood samples, the cattle were released. Genomic DNA was extracted from each blood sample and DNA sample quality was determined using an Agilent 2100 Bioanalyzer (Agilent, Santa Clara, CA, USA). The DNA fragment library of approximately 300 bp for each individual sample was constructed using the Wuhan Huada DNBSEQ sequencing platform. Whole-genome resequencing was performed with an average coverage of 10×. Each individual yielded an average of 29 GB of raw data, resulting in a total of 119,181,726 sequenced reads obtained. The total sequencing base count, which represents the total number of nucleotides sequenced, was an impressive 35,754,517,887. Quality control measures were used in the sequencing process, and an average of 97.62% of the bases in the sequencing reads had a mass value of 20 or above. GC content, or the percentage of guanine (G) and cytosine (C) bases in the DNA, is an important factor in genomic analysis and can provide insights into the structure and function of the genome. The average GC content of the sequenced DNA was determined to be 43.35%. In summary, the described sequencing process generated high-quality genomic data with substantial coverage, setting the stage for detailed genomic analyses and a deeper understanding of the genetic characteristics of the Hainan yellow cattle population.

### 2.3. Bioinformatics Analysis of Whole-Genome Sequencing Data

The raw data were merged and quality controlled (QC) using OpenGene, specifically using ‘fastp’, an ‘ultra-fast all-in-one’ FASTQ preprocessing software (https://github.com/OpenGene/fastp, version 0.20.1, accessed on 7 July 2022). All individual filtered reads were aligned to the cattle reference genome (ARSUCD1.2) by BWAMEN (V0.7.17) [12]. Sambamba (https://github.com/biod/sambamba, version 0.8.2, accessed on 15 July 2022) software was used to filter duplicate reads [13]. The original SNPs were called with GATK (V4.0.3.0) [14]. Genotype populating was performed using beagle, version beagle.22Jul22.46e.jar. ’HaplotypeCaller’, ’GenotypeGVCFs’, and ’SelectVariants’ are tools from GATK used to identify SNPs. HaplotypeCaller is a tool used to discover haplotypes in samples, representing genomic sequences at adjacent loci. It employs a re-assembly algorithm for more accurate variant detection, particularly in complex regions. GenotypeGVCFs is employed to merge haplotype calling files (gVCFs) and perform genotype calling for each sample. It transforms haplotype information into sample-level genotype information, providing genotypes and corresponding qualities at each variant locus. SelectVariants is used to choose specific types of variants or those meeting specific criteria from the called genotypes. As such, ‘SelectVariants’ serves to filter and select relevant variants for subsequent analysis. For controlling single nucleotide polymorphism (SNP) quality, VCFtools was utilized with the following parameters: ‘--minDP 4 --max-missing 0.5 --minQ 30 --maf 0.05 --minGQ 10′. Each parameter can be thought of as follows: (1) ‘--minDP 4’: sets the minimum depth, requiring a depth of at least 4 replicates at each variant locus; (2) ‘--max-missing 0.5’: sets the maximum deletion rate, requiring that the deletion rate of each mutation site does not exceed 0.5, that is, 50%; (3) ‘--minQ 30’: sets the minimum quality, requiring a quality value of at least 30 at each variant locus; (4) ‘--maf 0.05′: sets the minimum minor allele frequency, requiring a minimum minor allele frequency of 0.05, that is, 5% per mutation site; and (5) ‘--minGQ 10’: sets the minimum genotype quality, requiring a genotype quality of at least 10 at each variant locus [15].

### 2.4. Detection of Genome-Wide Selection Signatures

By detecting and analyzing runs of homozygosity (ROHs), we can uncover genetic differences among Hainan yellow cattle and other populations, as well as genetic variations that may impact specific traits or adaptability. ROHs can be used to identify inbreeding, determine selectively favored gene regions, and estimate genetic load. Long ROHs may indicate recent inbreeding, while short ROHs may represent distant common ancestors. This approach provides a robust foundation for understanding the genomic landscape, inbreeding patterns, and potential genetic factors influencing traits and adaptability in this specific cattle population. The PLINK (version V1.90) [16] software was used to identify runs of homozygosity (ROHs), by sliding a window of 50 SNPs across the genome (-homozyg-window-snp 50) to estimate/identify regions of repeated homozygosity. The following settings were used in our identification of ROH (setting choice in parentheses): (1) required minimum density (-homozyg-density 50); (2) number of heterozygotes allowed in a window (-homozyg-window-het 3); (3) the number of missing calls allowed in a window (-homozyg-window-missing 5). Whole-genome sequence data were used to characterize ROHs in Hainan yellow cattle. The use of whole-genome sequence data adds a comprehensive layer to the analysis, enabling a detailed characterization of ROHs in Hainan yellow cattle.

The basic idea underpinning composite likelihood ratio testing (the CLR method) is to compare the likelihood ratio between two hypotheses. In this case, one hypothesis assuming selection and the other assuming the absence of selection. By comparing the likelihood ratios under these two hypotheses, a statistical measure is obtained to assess whether the sampled genome has evidence of selection. CLR tests for sites in non-overlapping 50 kb windows were calculated using SweepFinder2 (version 2.1) [17]. In the context of population genetics, we performed CLR selection signal analysis to identify regions of the genome that exhibit abnormal levels of nucleotide diversity. Minor allele frequency differentiation between the five populations was used to create a model using Brownian motion to simulate neutral genetic drift [18]. In summary, CLR selection signal analysis involves comparing likelihood ratios under selection and non-selection hypotheses, specifically focusing on non-overlapping 50 kb windows. In the context of population genetics, it further considers multilocus allele frequency differentiation among populations and employs a model based on Brownian motion to simulate neutral genetic drift. The goal is to identify genomic regions exhibiting signals of selection and abnormal levels of nucleotide diversity, providing valuable insights into the genetic basis of traits such as heat tolerance in Hainan yellow cattle.

The fixed index was calculated by VCFTools, the genome was calculated by 20 kb steps using a 50 kb window, and the overall haplotype score was calculated using R (version 3.6.3) [19].

Tajima’s D is a statistical method used to detect signals of natural selection in the genome. It relies on two different measures of genetic polymorphism, namely single nucleotide polymorphism (SNP) and heterozygosity. By comparing the expected and observed values of these two measures, Tajima’s D can provide information about whether natural selection has occurred in the genome. Positive values of Tajima’s D may indicate less low-frequency variation, possibly due to positive selection, while negative values may suggest more low-frequency variation, potentially associated with population expansion or positive selection processes. This method is commonly employed in population genetics to analyze and identify signals of selection in the genome. The application of Tajima’s D is particularly useful for identifying candidate genes that have undergone selective pressures. In your context, the calculation of Tajima’s D statistic for each candidate gene is performed using VCFtools, a versatile software tool for working with Variant Call Format (VCF) files commonly used in genomic research. Overall, Tajima’s D provides a valuable framework for understanding the evolutionary forces shaping genetic diversity within populations and pinpointing genomic regions influenced by natural selection.

### 2.5. Functional Enrichment Analysis of Genes

In the context of this study, the identification of SNPs with selection signatures involved retrieving genetic information from the NCBI database, specifically utilizing the file GCF002263795.1ARS-UCD1.2_genomic.gff.gz. The subsequent step involved gene enrichment analysis, a crucial process that aids in comprehending the functions associated with a given gene set and their contributions to various biological processes. This analysis delves into the exploration of whether specific functions or pathways experience enrichment within a set of genes. Such enrichment can shed light on the collaborative actions of these genes in essential cellular processes, signaling pathways, or metabolic pathways. Understanding these interactions is pivotal for unraveling the intricacies of genetic mechanisms and their implications in the broader biological context. To gain deeper insights into the functionality and signal transduction pathways of the identified genes, the researchers employed DAVID (Database for Annotation, Visualization, and Integrated Discovery), accessible at http://david.ncifcrf.gov, accessed on 25 October 2023. This platform was utilized for conducting Gene Ontology (GO) term analysis and Kyoto Encyclopedia of Genes and Genomes (KEGG) pathways analysis. These analyses provide a systematic and organized framework for interpreting the roles of genes in terms of their involvement in biological processes and pathways. In the evaluation of statistical significance, a corrected *p*-value threshold of less than 0.05 was applied. This stringent criterion ensures that the observed enrichments or associations between genes and functional categories are not merely due to random chance but hold statistical significance.

## 3. Results

### 3.1. Selection Signatures and Candidate Genes under Selection

We compared whole-genome resequencing data from northern Chinese cattle (Yanbian cattle), central Chinese cattle (Nanyang and Qinchuan cattle), Indian Zebu cattle, and southern Chinese cattle (Hainan yellow cattle). After filtering, a total of 10,205,360 SNPs were retained for further analysis. The fixed index and composite haplotype score were calculated before the selective signal analysis (Supplementary Tables S2 and S3). Three methods, ROH, CLR, and Tajima’s D, were used to identify the top 1% of the empirical distribution of each statistical measure as the region to select for signal analysis.

**Figure 1:**
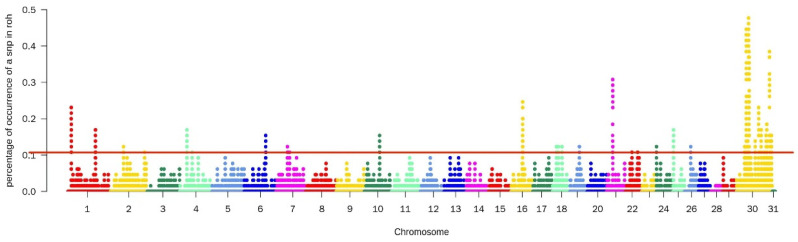
Manhattan diagram of the selective characteristics of ROH selective signatures. The solid red line represents the outlier classification threshold (top 5%). The Y-axis is the number of times an SNP occurred in an ROH, and the X-axis represents the position along each chromosome.

A total of 1290 SNPs were detected by the ROH method, mainly on chromosomes 21, 30, and 31. These particular chromosomes may have a higher prevalence of homozygous regions or regions with extended runs of homozygosity. After annotating these sites, 325 genes were identified (Figure 1).

**Figure 2:**
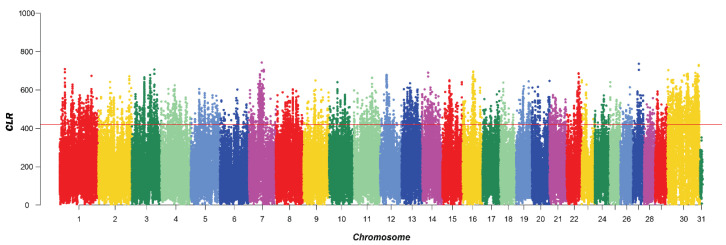
Manhattan plot of CLR selective signatures in the five breeds. The solid line illustrates the significant threshold level at a false discovery rate (FDR) of 5% (corrected *p*-value < 0.05). The Y-axis is the CLR, and the X-axis represents the position along each chromosome.

A total of 5210 SNPs were identified by CLR, mainly on chromosomes 1, 7, 8, and 30. This distribution suggested that these chromosomes may harbor regions with a higher density of genetic variation, which could be of interest for further genetic studies. A total of 453 genes were identified after annotation (Figure 2).

**Figure 3:**
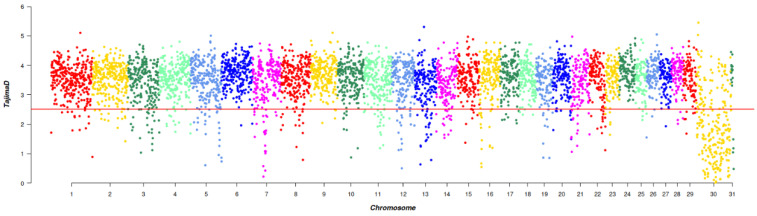
Manhattan plot of Tajima’s D selective signatures in the five breeds. The solid line illustrates the significant threshold level at a false discovery rate (FDR) of 5% (corrected *p*-value < 0.05). The Y-axis is the Tajima’s D value, and the X-axis represents the position along each chromosome.

A total of 1972 SNPs were detected by Tajima’s D, which were evenly distributed across the chromosomes. This uniform distribution suggested that they had experienced strong selection pressure or unusual genetic variation. Through Tajima’s D selection signal analysis, 450 genes were annotated (Figure 3).

**Figure 4:**
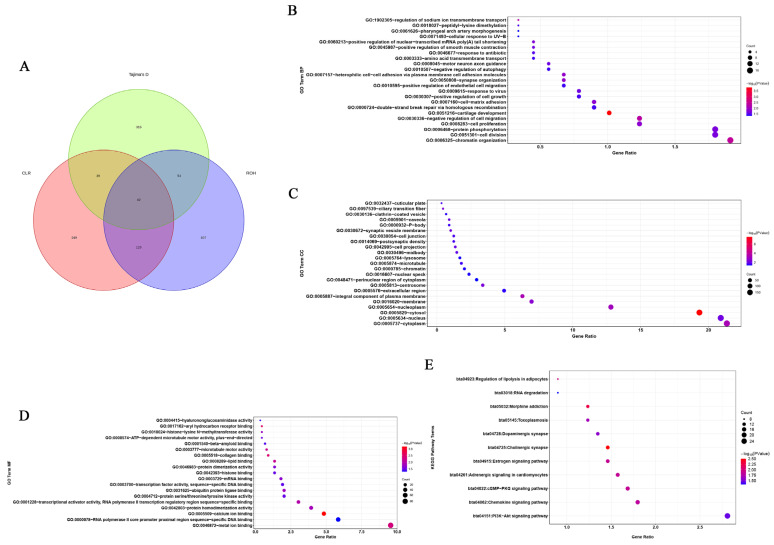
Venn diagram and bubble plot of Gene Ontology (GO) and Kyoto Encyclopedia of Genes and Genomes (KEGG) pathway analyses of candidate selection genes in cattle. (**A**) Venn diagram showing the overlap of candidate genes identified using each of the methods (ROH, CLR, and Tajima’s D). (**B**) The bubble chart of GO Biological Process (BP). (**C**) The bubble chart of GO Cellular Component (CC). (**D**) The bubble chart of GO Molecular Function (MF). (**E**) The bubble chart of KEGG pathway analysis of candidate selection genes.

We identified 453, 450, and 325 putatively advantageous positively selected genes from the CLR, Tajima’s D, and ROH methods, respectively (Figure 4A and Supplementary Table S4). In total, 42 genes were identified as being positively selected. This implies that these genes have undergone positive selection, suggesting that they may confer some adaptive advantages or play crucial roles in the evolutionary processes within the studied population. Positive selection on certain genes can indicate their importance in adaptation to specific environmental conditions, ecological niches, or other selective pressures. The diversity in the number of identified genes from different methods may stem from the distinct statistical approaches and assumptions underlying each method. Further analysis of these 42 positively selected genes could involve functional annotation to understand their roles and potential contributions to the adaptive traits observed. This information contributes to our understanding of the evolutionary dynamics and genetic diversity in the context of natural selection.

### 3.2. Functional Enrichment Analysis of the Candidate Genes in Five Breeds

After conducting enrichment analysis on the genes selected through the three aforementioned methods, 65 significant Gene Ontology (GO) terms and 11 significant KEGG pathways were revealed (Supplementary Table S5). These findings provide valuable insights into the molecular mechanisms and pathways involved in heat stress, and the identified candidate genes may play crucial roles in the cattle’s response to heat stress. Further research on these genes could contribute to a better understanding of heat stress adaptation and potentially lead to the development of strategies for managing heat stress in cattle populations. In the functional assignment of the significant GO terms suggested, ‘biological processes’ accounted for 35.38%, ‘cellular components’ represented 35.38%, and ‘molecular function’-related traits only 29.24%. The most significant GO term represented was the production of cytosol (GO:0005829—cytosol). Numerous GO terms associated with anti-heat stress and anti-inflammatory processes were significantly enriched, such as GO:0005634 (associated with the nucleus), GO:0005887 (associated with an integral component of the plasma membrane), GO:0005783 (associated with the endoplasmic reticulum), and GO:0005515 (associated with protein binding) (Figure 4B–D). The AMPK signaling pathway is a typical pathway associated with heat stress. Similarly, 11 significantly enriched pathways associated with heat stress were identified by KEGG analysis (Figure 4E). These included the following: GO:0005829—associated with cytosol, GO:0005887—associated with an integral component of the plasma membrane, GO:0005813—associated with the centrosome, GO:0005634—associated with the nucleus, GO:0031625—associated with ubiquitin protein ligase binding, GO:0000978—associated with RNA polymerase II core promoter proximal region sequence-specific DNA binding, bta04915—associated with the estrogen signaling pathway, and bta05145—associated with toxoplasmosis. Through a combined analysis of the functions of the 42 genes (Supplementary Table S6) identified by the aforementioned three methods and the pathway functions associated with these genes, five candidate genes related to heat stress were screened out, including *AHCYL2*, *DNAJC3*, *HSPA1A*, *CD53*, and *ZBTB12*. For *HSPA1A*, it has been reported that the expression of the gene was highly correlated with the temperature.

## 4. Discussion

Elevated temperatures can lead to physiological and molecular-level heat stress [20]. Hainan yellow cattle is a long-cultivated heat- and disease-tolerant breed distributed across a range of tropical and subtropical environments; this breed may be in a state of chronic heat stress. To investigate selection features in Hainan yellow cattle, three methods (ROH, CLR, and Tajima’s D) were employed to identify genetic variations and candidate genes associated with heat tolerance in this study; each method employed in this study relies on different statistical properties, providing a multifaceted approach to exploring the genomic landscape of Hainan yellow cattle. By elucidating the genetic mechanisms underlying heat adaptation in this breed, this study contributes valuable information for breeding programs aimed at enhancing heat resilience in livestock, particularly in the context of changing climates and environmental challenges.

Heat stress usually involves a series of biological processes, such as protein folding, antioxidative reactions, cellular apoptosis, and inflammation, among others [21,22,23,24,25,26]. Enrichment analysis was also conducted on the genes selected through these three methods. This study delves into the molecular and genetic aspects of heat stress, shedding light on the specific biological processes and pathways involved. The identification of enriched terms and genes provides valuable insights into the mechanisms underlying heat stress response and potential targets for further research or applications in enhancing heat stress resistance. Among the 65 GO terms, 6 enriched terms were potentially related to heat stress. Furthermore, we identified 11 enriched genes among known KEGG pathways, 2 of which are associated with heat stress resistance.

The AMPK signaling pathway is involved in a variety of cellular processes, including energy homeostasis, mitochondrial autophagy, and anti-inflammatory responses. Heat stress is often associated with apoptosis and inflammation. The AMPK signaling pathway responds to some stressful situations by inducing mitochondrial autophagy, which plays a crucial role in the cellular response to heat stress [27]. The AMPK pathway has also been shown to have a protective effect against oxidative stress-induced glomerulonephritis, which also plays an important role in the fight against cellular inflammation [28]. The cGMP-PKG signaling pathway affects platelet release, and the platelet content is related to inflammatory heat stress, indicating that this pathway is related to heat stress to a certain extent [29]. The AMPK signaling pathway may induce mitochondrial autophagy, and the cGMP-PKG signaling pathway may change the content of platelets to counter the inflammatory response in the body, so that Hainan yellow cattle can adapt to a hot climate.

To investigate the function of these genes, we compared the selected regions with previously annotated genes and observed strong differences in the signals in regions containing known candidate genes associated with heat tolerance. Five candidate genes that may be related to heat tolerance were identified. The genes were *AHCYL2*, *DNAJC3*, *HSPA1A*, *CD53*, and *ZBTB12*.

CD53 is an inhibitory factor that suppresses the production of inflammatory cytokines, integrating inflammatory and metabolic signals to respond to the nutritional status of liver cells. Blocking *CD53* may be a means to alleviate diseases associated with overeating and inflammation [30,31]. CD53 has the ability to protect mutated hematopoietic stem cells (HSCs) from inflammation and proliferative stress [32]. The *CD53* gene is also associated with the apoptotic pathway [33], and apoptosis is associated with heat stress. Additionally, the apoptotic pathway associated with heat stress may make *CD53* a factor involved in responding to environmental pressure and heat stress. It is possible, therefore, that *CD53* might contribute to the adaptation of Hainan cattle to the local climate by inhibiting the inflammatory response.

Expression of ZBTB12 is associated with lowered white blood cell counts and increased/decreased clotting time. White blood cells play a central role in the body’s inflammatory response [34]. *ZBTB12* is a gene associated with coagulation and inflammation [35]. This gene was identified in Hainan yellow cattle, and we suggest expression of this gene in the breed may indicate that Hainan yellow cattle mitigate the inflammatory response caused by heat stress by lowering white blood cell counts.

Similarly, genetic variations in the *AHCYL2* gene significantly affect fenofibrate therapy, and changes in the IL-6-related inflammatory pathways may be key mediators of the fenofibrate response. This finding indicates that there is a connection between the *AHCYL2* gene and internal inflammatory processes [36]. In hot areas, the body’s inflammatory response is usually triggered by heat stress [37]. The *AHCYL2* gene may also be involved in inflammatory processes in Hainan yellow cattle, helping to counteract the hot climate in Hainan by reducing the body’s inflammatory response.

DNAJC3 is a typical member of the DNAJ family. DNAJ proteins are molecular chaperone proteins that play a crucial role in the cellular heat stress response. *DNAJC3* in particular is known to be a powerful anti-inflammatory [38]. The activation of pro-inflammatory, autophagy, and apoptosis genes is a characteristic of ulcerative colitis, similar to the features of heat stress. DNAJC3 can also suppress the inflammatory factors produced in ulcerative colitis [39]. The GO:0005829—cytosol pathway is involved in heat stress, and the *DNAJC3* gene is also involved in this pathway. The *DNAJC3* gene may play a crucial role in the anti-inflammatory pathways of Hainan yellow cattle.

The four genes *AHCYL2*, *DNAJC3*, *CD53*, and *ZBTB12* may inhibit the inflammatory response in the body of Hainan yellow cattle in combination to resist the heat stress.

HSPA1A is a member of the heat shock protein family A (Hsp70). When bound to other heat shock proteins, these proteins can stabilize aggregates of existing proteins and mediate the folding of newly translated proteins in the cytoplasm and organelles. The expression levels of HSPA1A increased in heat-resistant mouse epidermis and HaCaT cells, and it has been established that HSPA1A plays a crucial role in mitigating the impact of elevated temperatures on cellular function [40]. In fact, applied genetic research has shown that expression of the *HSPA1A* gene can be used to suppress mastitis in Chinese Holsteins [41]. HSPA1A has a neuroprotective effect on TSCI and may inhibit apoptosis by activating the Wnt/β-catenin signaling pathway [42]. The expression of *HSPA1A* varies significantly across different breeds and different Temperature–Humidity Index (THI) conditions. Vechur cattle, adapted to the prevailing high temperature and humidity in the breeding grounds of Kerala, India, show significant expression of *HSPA1A* compared to *b.t. taurus*. This suggests that *HSPA1A* is a useful marker for selecting heat-tolerant animals [43]. Therefore, this gene may reduce cellular damage from heat stress during the immune response of Hainan yellow cattle, enabling them to adapt to hot environments.

The Hainan yellow cattle breed is a valuable genetic resource because of its associated heat, disease, and roughage resistance, and it is suitable for widespread breeding in tropical areas. As heat stress management continues to evolve, the genetics of cattle are also changing through continuous selection in breeding programs. Therefore, traditional breeding can produce different breeding methods [44]. Compared with traditional breeding methods, the advantage of genome selection is to improve the selection accuracy [45]. Therefore, it is most appropriate to use the candidate genes that have been screened in this study to breed more heat-resistant cattle breeds at the genome level.

This study identified pathways and candidate genes associated with heat stress. The selection pressure on these genomic regions related to heat may explain the well-established tolerance of temperatures associated with the species. This may, in part, explain why Hainan yellow cattle are known to be extremely heat tolerant.

## 5. Conclusions

Selection has left important footprints throughout the Hainan yellow cattle genome. In this study, five candidate genes were identified by selective signal analysis, which may explain the heat tolerance associated with Hainan yellow cattle. This study may be helpful for developing further heat resistance in cattle and provide the basis for the identification of more heat-related genes, further adaptation research, resource conservation, and breeding improvement of Hainan yellow cattle in the future.

## Data Availability

The data were deposited in the National Center for Biotechnology Information’s Short Read Archive BioProject repository, under the accession number PRJNA967096.

## Supplementary Materials

The following supporting information can be downloaded at: https://www.mdpi.com/article/10.3390/ani14050775/s1, Table S1: additional cattle sample table for selection signal analysis of Hainan yellow cattle; Table S2: the score of fixed index; Table S3: the score of composite haplotype; Table S4: a summary table of the genes screened out separately by ROH, CLR, and Tajima’s D; Table S5: the results of GO and KEGG enrichment analysis; Table S6: summary of genes collectively screened by three methods (ROH, CLR, and Tajima’s D).
